# Does the information in the phase of low frequency LFP reflect the low frequency envelope of local spike rates?

**DOI:** 10.1186/1471-2202-12-S1-P227

**Published:** 2011-07-18

**Authors:** Sohail Siadatnejad, Stefano Panzeri, Christoph Kayser, Nikos K Logothetis, Marcelo A Montemurro

**Affiliations:** 1 Faculty of Life Sciences, University of Manchester, Manchester, M13 9PT, UK; 2 Robotics, Brain and Cognitive Sciences Department, Italian Institute of Technology, Genoa, 16163, Italy; 3 Max Planck Institute for Biological Cybernetics, Tübingen, 72076, Germany; 4 Imaging Science and Biomedical Engineering, University of Manchester, Manchester M13 9PT, UK

## 

Recently, it has been shown that when the timing of spikes is measured relative to the phase of the cortical local field potentials (LFP), spikes can carry substantial more information about an external stimulus [1]. Experimental studies in sensory cortices of macaque have shown that the extra information obtained with such *phase-of-firing* codes above that in the firing rate alone ranges from 55% in primary visual cortex [1] to more than 100% in primary auditory cortex [2]. Here, we use a mathematical model that relates local spike trains and the resulting LFP, to explain the emergence of the phase-of-firing codes in cortex. The model is based on the one proposed in [3] and incorporates two types of integration over the spiking activity: i) a time convolution that results from the filtering properties of neural structures [4], which embeds history effects in LFP from past spiking activity, and ii) an integration step over the activity of neurons in the neighbourhood of the measuring electrode.

**Figure 1:**
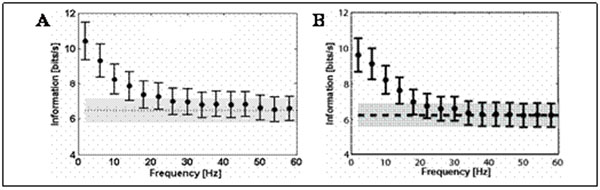
Comparison of the phase-of-firing information using synthetic and real LFP**.** The original data corresponds to LFP and spiking activity from 78 recordings channels in macaque V1, obtained while the animals were presented a movie [1]. **A.** Information in the phase-of-firing code as a function of the LFP frequency band (black dots, with error bars indicating SEM over the dataset), and in the spike count (dashed line, with SEM indicated by grey area). In this panel, the LFP was simulated using real spikes with a mathematical model based on the one in [3]. **B.** As in **A**, however in this case both spikes and LFP correspond to real data [1].

When the spikes recorded from macaque primary visual cortex were used to synthesize the LFP, the model could reproduce the phase-of-firing information found using the real LFP, as shown in Figure 1. This suggests that an important component of phase-of-firing information originates from the surrounding neural population and past spiking activity. The next question that arises is what is the relative contribution of the neuron population size and the length of the firing rate history embedded in the LFP. We are currently investigating this question by parametrically varying both the population size and time integration ranges in generating the synthetic LFP.
